# Performance of Cement Paste with Denitrified Fly Ash Containing NH_4_HSO_4_

**DOI:** 10.3390/ma15176083

**Published:** 2022-09-02

**Authors:** Yuan Wang, Zhi Wang, Hongyi Qin, Linbo Jiang, Jinghang Niu, Zhenhua Liu

**Affiliations:** 1 College of Material Science and Engineering, Chongqing University, Chongqing 400045, China; 2 Chongqing Academy of Metrology and Quality Inspection, Chongqing 400045, China; 3 College of Water and Architectural engineering, Shihezi University, Shihezi 832000, China

**Keywords:** denitrified fly ash, hydration, volume change, NH_4_^+^, ammonia content

## Abstract

The denitrification process was completed in coal-fired power plants, resulting in the fly ash containing NH_4_HSO_4_. When this kind of fly ash with ammonia was applied to cement and concrete, there could be phenomena such as a retarded setting time, decreased compressive strength, and volume expansion. This paper mainly investigated the influence of fly ash containing NH_4_HSO_4_ on the properties of fly ash cement paste, and pastes with NaHSO_4_ were set as the control samples. The research on Na^+^ in cement hydration was studied. The influence of NH_4_HSO_4_ content in fly ash on the properties of fly ash cement paste was also investigated. It was found that NH_4_^+^ could greatly affect the properties of fly ash cement paste, such as significantly reducing the fluidity, prolonging the setting time, decreasing the compressive strength, increasing the drying shrinkage, decreasing the total heat released during the hydration, and affecting the content of calcium hydroxide hydrate. Increasing the ammonia content of the denitrified fly ash would reduce fluidity, retard its setting time, increase the porosity of the cement stone, and increase the number of pores with large sizes in the fly ash cement paste. The increase of porosity and pores with large sizes in cement decreases the compressive strength and increases the drying shrinkage of the fly ash cement paste.

## 1. Introduction

Coal produces large amounts of nitrogen oxides and CO_2_ during combustion, resulting in a significant amount of environmental pollution [1,2]. Coal-fired power plants in China have completed the denitrification process, so the fly ash in China is denitrified fly ash containing ammonium salt. Ammonia slip is inevitable in the process of flue gas denitrification, and ammonium salt will be engendered and adsorbed on the fly ash particles due to the reaction of escaping NH_3_ with the SO_3_ of the flue gas. As a result, the problem of the utilization of denitrified fly ash is becoming more and more common in concrete. A large number of studies have shown that NH_4_HSO_4_ is a by-product of denitrified fly ash [3,4,5,6,7,8,9].

Denitrified fly ash has many problems when used in building materials. Wu [10] found that the ready-mixed concrete with the denitrified fly ash caused uneven bubbles after the concrete was poured and bubble marks in concrete after the concrete hardened. Schert et al. [7] showed that the release rate of NH_3_ gradually decreased to the level of undetectable in concrete with denitrified fly ash after three weeks of continuous release; however, a large amount of ammonia (20–70% relative to the initial level) remained in the concrete. He [11] showed that the ammonia of denitrified fly ash could influence the macroscopic properties of the fly ash cement paste, such as reduced rheological properties and decreased mechanical properties. Tan et al. [12] showed that denitrified fly ash can prolong the setting time and reduce the compressive and flexural strength of fly ash cement paste. Therefore, the application of denitrified fly ash in concrete will have many problems, such as prolonging the setting time [13], increasing the loss of fluidity [14], the appearance of a large number of bubbles and irritating odor on the surface of the concrete [15], leaving holes on the surface of the hardened concrete, causing the expansion of the concrete volume and decreasing the compressive strength [16], serious surface pulverization, etc. The problem of denitrified fly ash in the application of cementitious materials may be caused by the reaction between NH_4_^+^ and OH^−^ in the hydration process of cement to release ammonia and reduce the alkalinity of the cement paste. At present, there are few studies on the influence of NH_4_^+^ on the properties of fly ash cement paste under the control of the anions (HSO_4_^−^).

The ammonia content in denitrified fly ash is also worthy of attention. According to the literature, the ratio of ammonia content adsorbed on fly ash (calculated from the mass fraction of NH_3_) to ammonia concentration that escapes from the flue gas is around 50:1, and the ammonia content in denitrified fly ash ranges from 0–2500 ppm [7]. Zhang [17] collected and tested the nitrogen content of 30 fly ash samples from 15 power plants, and the results revealed that the ammonia content of most fly ash samples collected is less than 100 ppm. The ammonia content used in the study about denitrified fly ash was 0.1–6% [18,19], which differs greatly from the truly denitrified fly ash. In addition, the release of ammonia inevitably leads to changes in volume. However, there are few studies on the influence that ammonia content has on the volume of fly ash cement paste.

In this paper, the effect of Na^+^ on cement hydration was widely investigated in the literature, so the effect of NH_4_^+^ and Na^+^ on the performance of fly ash cement paste was compared while the anions (HSO_4_^−^) were held constant. In addition, the performance of cement paste was studied with the fly ash of different ammonia content, such as setting time, compressive strength, drying shrinkage, and pore structure. The ammonia content in fly ash was varied with the addition of NH_4_HSO_4_. These studies will provide a reference for the subsequent application of denitrified fly ash in cement.

## 2. Raw Materials and Test Methods

### 2.1. Raw Materials

The fly ash used in the experiment was obtained from a power plant in Chongqing. The content of the fly ash was 30% in the fly ash blended cements. The initial ammonia content of the fly ash was 105 mg/kg, as determined by the fly ash rapid ammonia analyzer. The specific surface area of the fly ash was 434 m^2^/kg. The cement used in the experiment was 42.5R ordinary Portland cement produced by the Chongqing Xiaonanhai Cement Plant. The specific surface area of the cement was measured as 375 m^2^/kg by Brunauer–Emmett–Teller surface area measurement. The chemical composition of the fly ash and cement is listed in Table 1. Standard sand produced by Xiamen Aisiou Standard Sand Co., Ltd. (Xiamen, China) was used in the experiments. The density of the sand was 1.610 g/cm^3^, and the content of SiO_2_ was 98.5%, which meets the GB/T17671-1999 “Method of testing cements -Determination of strength”. The chemicals used in the experiment were analytically pure, mainly ammonium bisulfate (NH_4_HSO_4_), sodium bisulfate (NaHSO_4_), and ethanol. Tap water was used for the preparation the samples for the experiment, which is in accordance with the standard for water in concrete (JGJ63-2006). Deionized water was used for chemical analysis. The detailed mix proportions to compare the effects of NH_4_HSO_4_ and NaHSO_4_ are presented in Table 2. The mixed proportions of ammonia content are presented in Table 3.

### 2.2. Methods

The preparation and testing of the fluidity conformed to GB/T8077-2012. As the sample did not use additives, the water–binder ratio was 0.5. The setting time was determined using the Vicat apparatus following the Chinese standard procedure GB/T1346-2011, and the water–binder ratio was 0.4. The compressive strength was measured in accordance with the standard GB/T17671-1999, and the water–binder ratio was 0.4. The pressure testing machine was the TYE-300B produced by Wuxi Jianyi Instrument Machinery Co., Ltd. (Wuxi City, China).

According to the drying shrinkage test reference standard JC/T603-2004, the water–binder ratio was 0.4. The fresh paste was cast into 25 mm × 25 mm × 280 mm metal cube molds and vibrated for 2 min to remove large air bubbles. After 24 h, the specimens were removed from the molds and the initial length *L*_0_ measured. The specimens were cured in a room at a temperature of 20 ± 2 °C with a relative humidity of 65 ± 5% until testing. The length (*L_t_*) of the specimen at different times was measured using a micrometer, and the drying shrinkage at different times was calculated using Equation (1):

*St* = ((*L*_0_ − *L_t_*) × 100)/250 (1)

where *S_t_* is the drying shrinkage rate of the cement specimen at *t* days, *L*_0_ is the initial length of the specimen, *L_t_* is the length of the specimen after *t* days, and 250 is the effective length of the specimen.

The hydration heat of the fly ash cement paste was tested using a TAM Air 8 channel microcalorimeter produced by TA Instruments. The water–binder ratio was 0.4; the temperature of the water was adjusted to 20 °C; the duration of the test was 3 days.

The specimens cured to ages were broken apart, and the center of the specimens was collected in the form of small pieces. The hydration of the crushed solid was terminated by immersion in absolute ethyl alcohol and further vacuum drying at 40 °C to remove the solvent. Then, the samples were ground and sieved to 75 μm before the X-ray diffraction (XRD) and TG-DTG measurements. During the XRD test, the step length was set to 0.02°, and the scanning time was 10 min. TG-DTG was used to analyze the change in mass of the cement hydration products during heating. The samples were heated from 30–1000 °C in a nitrogen atmosphere at a rate of 10 °C/min.

The pore structure of the fly ash cement paste was tested using the mercury intrusion method; measurements were conducted using an AutoPore IV 9500 high-performance automatic mercury intrusion instrument produced by Micromeritics.

## 3. Results and Discussion

### 3.1. Properties of the Cement Paste

#### 3.1.1. Fluidity

Fly ash contains spherical particles with smooth surfaces and are smaller than the cement particles; thus, fly ash has both a morphological effect and micro-aggregate filling effect, which improve the workability, reduce water demand, and improve the fluidity of the mixture when used as mineral admixtures in cement and concrete [20]. However, the presence of the denitrification by-product NH_4_HSO_4_ may instead reduce both the fluidity and fluidity loss of the fly ash cement paste when denitrified fly ash is used as a mineral admixture.

**Figure 1:**
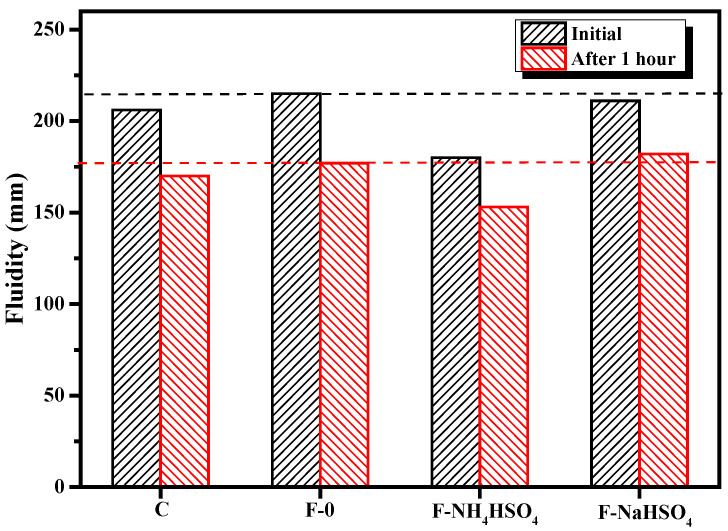
Effect of different sulfates on the fluidity of fly ash cement paste.

The effect of NH_4_HSO_4_ and NaHSO_4_ on the initial fluidity and fluidity after 1 h of the fly ash cement paste is shown in Figure 1. Fly ash without the addition of NH_4_HSO_4_ and NaHSO_4_ can improve the fluidity of the fly ash cement paste. However, the initial fluidity of the fly ash cement paste was reduced by 17.0% and 2.7%, when 2% NH_4_HSO_4_ and 2% NaHSO_4_ were added to the fly ash, respectively. This suggests that the influence of NH_4_HSO_4_ on the fluidity of the fly ash cement paste is relatively large and implies that NH_4_^+^ may affect the fluidity of the fly ash cement paste. Possible reasons for these observations are: (1) NH_4_^+^ reacts with OH^–^ in water to generate NH_3_·H_2_O after ammonium bisulfate is dissolved in water, which consumes the water in the paste and, thus, reduces its fluidity; (2) NH_4_^+^ in ammonium bisulfate reacts with the Ca(OH)_2_ generated during the early hydration of the cement to generate NH_3_·H_2_O, which results in the release of ammonia. Ammonia will dissolve in water to produce NH_3_·H_2_O and consume part of the water, decreasing the amount of water in the paste and, consequently, decreasing its fluidity. In summary, NH_4_^+^ mainly affects the fluidity of the paste when denitrified fly ash is used in the cement paste.

#### 3.1.2. Setting Time

Fly ash can retard the setting time of the fly ash cement paste. The research on the influence of sulfate on cement hydration is as follows: gypsum (calcium sulfate) can adjust the setting time of cement paste; sodium sulfate is an early strength accelerator for cement that can promote early hydration. Alkali sulfates also have a significant influence on the setting time of cement. Hence, it is worth discussing the influence that NH_4_HSO_4_ and NaHSO_4_ have on the setting time of the fly ash cement paste.

**Figure 2:**
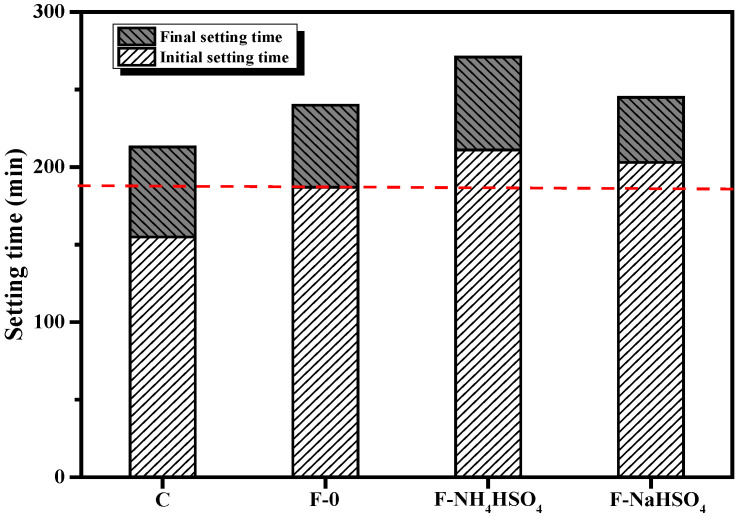
Effect of different sulfates on the setting time of fly ash cement paste.

Studies have shown that NH_4_HSO_4_ in denitrified fly ash has a certain retarding effect [21,22]. This is possibly related to the higher concentration of sulfate ions introduced by NH_4_HSO_4_ [19]. The influence of NH_4_HSO_4_ and NaHSO_4_ on the setting time of the fly ash cement paste is shown in Figure 2. Both NH_4_HSO_4_ and NaHSO_4_ retard the setting time, whereas the latter has a much smaller effect, which suggests that NH_4_^+^ greatly slows down the hydration of the fly ash cement paste.

#### 3.1.3. Compressive Strength

When denitrified fly ash is used as a mineral admixture in cement and concrete engineering, different degrees of decline in compressive strength are observed. When NH_4_HSO_4_ in denitrified fly ash is used as a sulfate, it could excite the activity of the fly ash and improve the strength of the fly ash cement paste. However, the existence of NH_4_^+^ leads to the release of ammonia and affects the density of the cement stone, which may have a negative impact on the mechanical properties of the fly ash cement paste.

**Figure 3:**
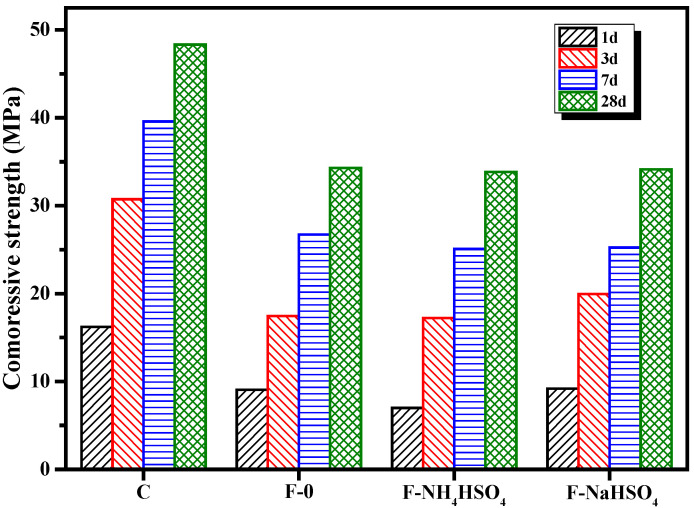
Effect of different sulfates on the compressive strength of fly ash cement paste.

Figure 3 shows the effect of NH_4_HSO_4_ and NaHSO_4_ on the compressive strength of the fly ash cement paste. The results showed that NH_4_HSO_4_ can reduce the compressive strength of the fly ash cement paste, and the compressive strength of 1 d was 22.8% lower than that of the fly ash cement paste without NH_4_HSO_4_. The compressive strength of the fly ash cement paste with NaHSO_4_ was 1.3% and 14.3% higher than the samples without NaHSO_4_ after 1 and 3 days, respectively, while the compressive strengths of the samples after 7 and 28 days were effectively identical. This suggests that NH_4_^+^ reduces the compressive strength of the fly ash cement paste.

#### 3.1.4. Drying Shrinkage

The mixing of fly ash into cement paste will reduce its drying shrinkage [20]. However, NH_4_^+^ ions contained within NH_4_HSO_4_ present in denitrified fly ash may react with OH^–^ in both the water and calcium hydroxide generated by cement hydration to release ammonia, which can influence the drying shrinkage of the fly ash cement paste.

**Figure 4:**
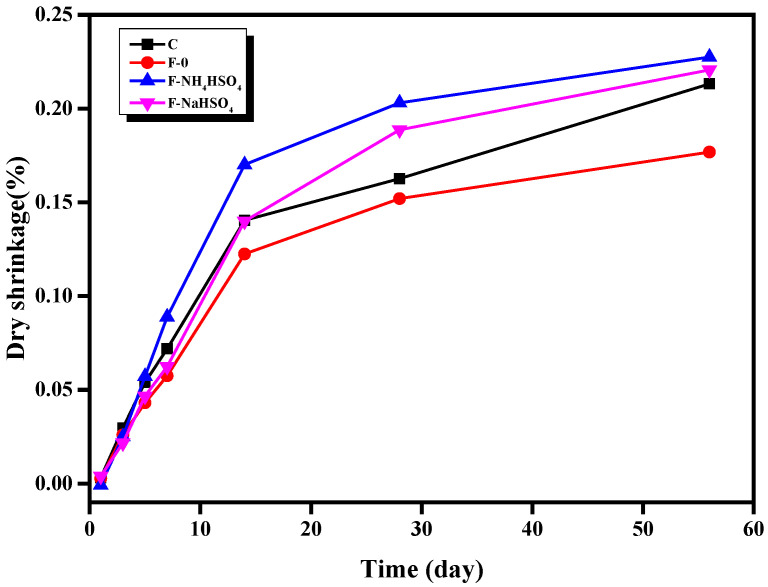
Effect of different sulfates on the drying shrinkage of fly ash cement paste.

The effects of NH_4_HSO_4_ and NaHSO_4_ on the drying shrinkage of the fly ash cement paste are shown in Figure 4. Without the addition of sulfate, fly ash can reduce the drying shrinkage of the fly ash cement paste, while the addition of NH_4_HSO_4_ and NaHSO_4_ results in a clear increase in the drying shrinkage of the fly ash cement paste. In addition, the drying shrinkage of the fly ash cement paste containing NH_4_HSO_4_ was larger than that of the samples that contained NaHSO_4_, indicating that NH_4_^+^ has a greater influence on the drying shrinkage. One possibility is that NH_4_HSO_4_ in denitrified fly ash reacts with water, and the volatilization of NH_3_·H_2_O also removes the water, increasing the drying shrinkage. The volatilization of NH_3_ itself also can cause the contraction of the paste. Another possibility is that the release of ammonia causes the opening porosity of the hardened fly ash cement paste to increase, which accelerates the loss of water and leads to drying shrinkage. Therefore, NH_4_^+^ in denitrified fly ash will increase the drying shrinkage of the fly ash cement paste.

#### 3.1.5. Heat of Hydration

The addition of fly ash reduces the exothermic peak of cement hydration and delays the time at which this peak occurs. As a sulfate, NH_4_HSO_4_ may also affect the hydration heat of the fly ash cement paste. Therefore, the effects of NH_4_HSO_4_ and NaHSO_4_ on the hydration heat of the fly ash cement paste were studied.

**Figure 5:**
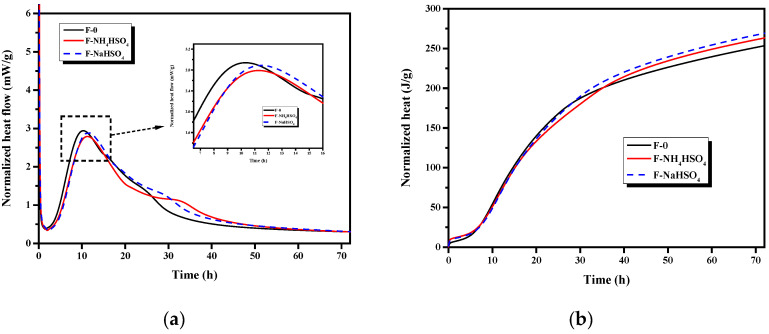
Effect of different sulfates on (**a**) heat flow curve and (**b**) cumulative heat release of the cement fly ash system.

The effect of NH_4_HSO_4_ and NaHSO_4_ on the heat of hydration of the fly ash cement paste is shown in Figure 5. The hydration heat peak of the fly ash cement paste containing NH_4_HSO_4_ and NaHSO_4_ was observed to be lower than that of the samples without sulfate, and the peak of the hydration heat was delayed; notably, the peak hydration heat reached by samples containing NaHSO_4_ was higher than that of those that contained NH_4_HSO_4_. This indicates that both NH_4_HSO_4_ and NaHSO_4_ slow down the process of cement hydration, but NH_4_HSO_4_ results in longer delays. In terms of the total amount of heat released, the heat of hydration released by the fly ash cement paste containing NH_4_HSO_4_ and NaHSO_4_ was slightly lower than samples without sulfate in the first 30 h; however, the total heat release was greater after this time had elapsed. This suggests that NH_4_HSO_4_ and NaHSO_4_ reduce the total hydration heat of the fly ash cement paste in the first 30 h of hydration. After 30 h, the sulfates may stimulate the activity of the fly ash, promoting the secondary hydration of the fly ash and increasing the total hydration heat of the fly ash cement paste.

#### 3.1.6. Hydration Products

C-S-H, Ca(OH)_2_, and other hydration products are generated when cement reacts with water. The types of cement hydration products are closely related to their mechanical properties and volume stability. The addition of NH_4_HSO_4_ and NaHSO_4_ may affect the hydration products of the fly ash cement paste. The effects of NH_4_HSO_4_ and NaHSO_4_ on the hydration products of the fly ash cement paste at different ages were studied by XRD and TG.

**Figure 6:**
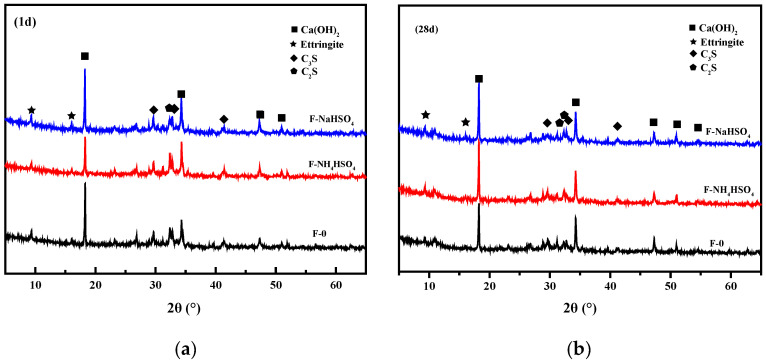
XRD patterns of the hydration products of the cement fly ash paste after (**a**) 1 day and (**b**) 28 days.

The XRD patterns of the hydration products of the fly ash cement paste with NH_4_HSO_4_ and NaHSO_4_ after 1 day and after 28 days is shown in Figure 6. NH_4_HSO_4_ and NaHSO_4_ do not affect the types of hydration products of the fly ash cement paste at different ages, i.e., no new hydration products were generated, indicating that NH_4_HSO_4_ and NaHSO_4_ do not affect the hydration products generated in the fly ash cement paste. In addition, a semi-quantitative analysis of the hydration products was carried out according to the diffraction peaks. After one day, the diffraction peaks associated with calcium hydroxide in the samples containing NH_4_HSO_4_ and NaHSO_4_ were relatively low, which may be due to the consumption of calcium hydroxide by NH_4_^+^ and H^+^. There was no significant difference in the amount of calcium hydroxide in the samples after 28 days, indicating that the NH_4_HSO_4_ and NaHSO_4_ mainly affect the concentration of calcium hydroxide in the early stages of hydration.

**Figure 7:**
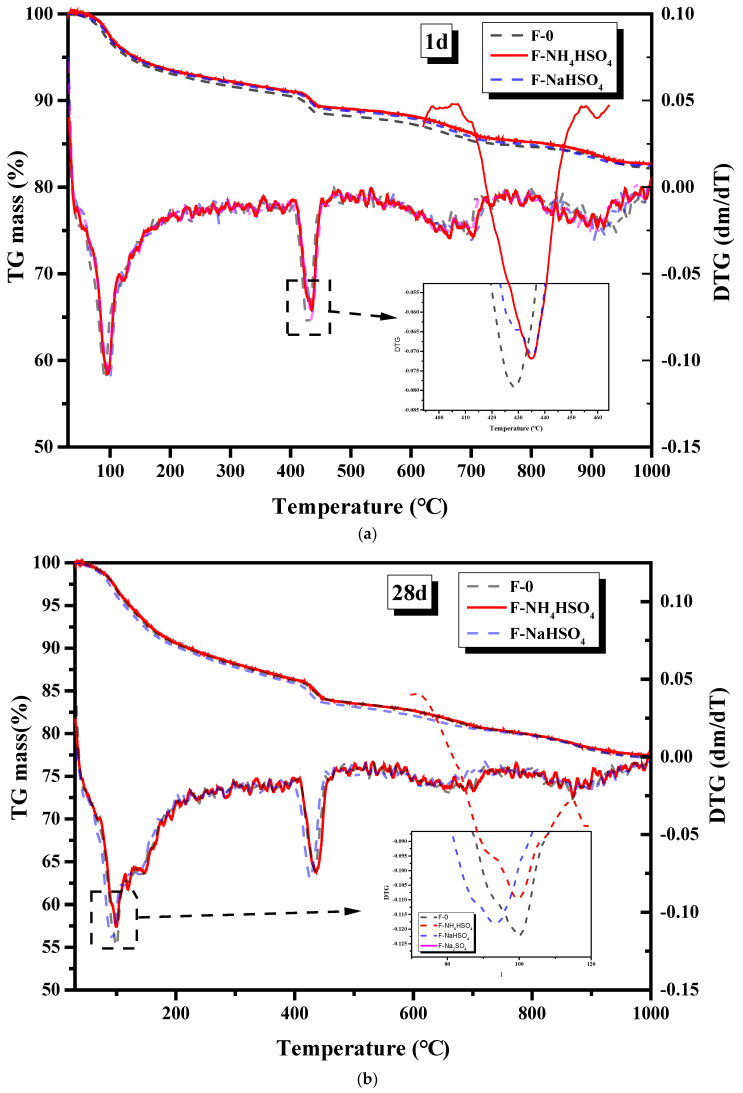
TGA spectra of the hydration products of the fly ash cement paste after (**a**) 1 day and (**b**) 28 days.

The TGA spectra that describes the influence of NH_4_HSO_4_ and NaHSO_4_ on the hydration products of the fly ash cement paste after 1 d and 28 d is shown in Figure 7. The decomposition peak of ettringite and C-S-H gel was at 70–100 °C, while the decomposition peak of calcium hydroxide occurred between 420 and 475 °C [23]. The calcium hydroxide production in the cement paste samples that contained NH_4_HSO_4_ and NaHSO_4_ was observed to be lower than that in samples without sulfate after one day, which is consistent with the XRD test results, supporting the theory that NH_4_^+^ and H^+^ consume a portion of the available calcium hydroxide. Furthermore, at 1 day, the reactivity of the cement was delayed in the presence of NH_4_HSO_4_, so less portlandite was produced. After 28 days, there was no difference in the amount of calcium hydroxide present in the samples containing NH_4_HSO_4_ and the samples containing NaHSO_4_.

### 3.2. Properties of the Fly Ash Cement Paste with Additional NH_4_^+^

The results obtained in Section 3.1 indicate that the NH_4_^+^ in NH_4_HSO_4_ reduces the fluidity of the fly ash cement paste, retards its setting time, decreases its compressive strength, and increases its drying shrinkage, but does not affect the hydration products produced. However, the ammonia content of 2% NH_4_HSO_4_ and actual denitrified fly ash (0–2500 ppm) is quite different [16], and there have been few studies on the effects of ammonia content on the volume and pore structure of fly ash cement paste. Thus, the study of the effects of ammonia content on the volume change of cement-based materials is of practical significance.

#### 3.2.1. Fluidity

**Figure 8:**
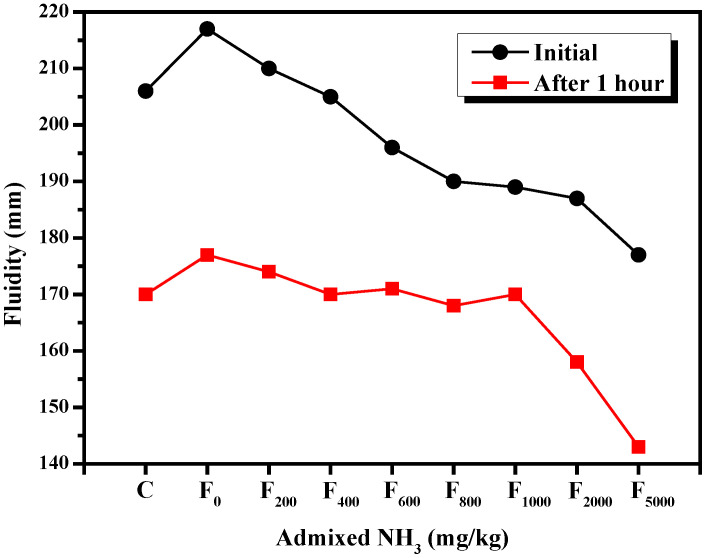
Changes in the fluidity of fly ash cement paste with increasing ammonia content.

The results presented in Section 3.1.1 showed that NH_4_^+^ reduces the initial fluidity of the fly ash cement paste and increases the fluidity loss. The change in the fluidity of the fly ash cement paste in the samples with varying ammonia contents is shown in Figure 8. It can be seen that the fluidity of the fly ash cement paste increases with the increase of ammonia content. When no ammonia was added to the fly ash, the initial fluidity of the fly ash cement paste was 5.3% higher than that of the cement paste. However, as the additional ammonia content increased, the fluidity of the fly ash cement paste decreased significantly. When the additional ammonia content was 200 mg/kg, the initial fluidity of the fly ash cement paste was still 2% greater than that of cement paste. However, when the additional ammonia content in the fly ash was 400 mg/kg, the initial fluidity of the fly ash cement paste was equivalent to that of the cement paste. Finally, when the additional ammonia content of the denitrified fly ash exceeded 400 mg/kg, the fluidity of cement paste was reduced rather than increased.

In addition, the fluidity loss of the samples after the first hour decreased and then increased. When the additional ammonia content was less than 1000 mg/kg, the fluidity loss after the first hour exhibited a decreasing trend. Notably, the fluidity loss after the first hour of the fly ash cement paste when 1000 mg/kg ammonia was added was 52.5% lower than that of the sample without any added ammonia. One possibility was that the release of ammonia led to the emergence of small bubbles in the fly ash cement paste, which potentially lubricated the paste. When the additional ammonia content exceeded 1000 mg/kg, the fluidity loss increased over time; specifically, the one-hour fluidity loss in the sample with 5000 mg/kg of additional ammonia was 79% greater than in the sample where only 1000 mg/kg ammonia was added. This could be because ammonia was very soluble in water when the paste was saturated with ammonia, and it will form NH_3_·H_2_O with the water in the paste. In addition, the volatilization of NH_3_·H_2_O will also consume some of the water in the system. This negative effect of the loss of water on the fluidity of the fly ash cement paste is greater than the increase in fluidity provided by the bubbles generated by this process, resulting in the overall fluidity loss of the fly ash cement paste over time.

#### 3.2.2. Setting time

**Figure 9:**
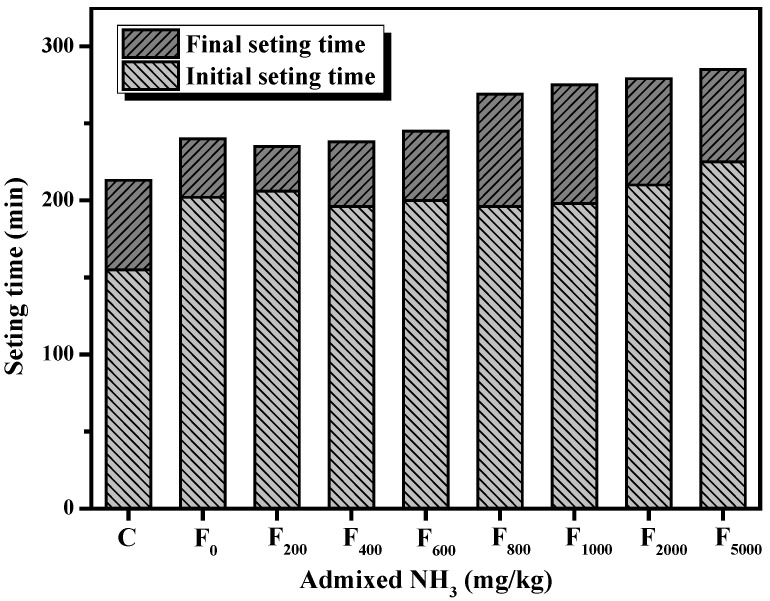
Changes in the setting time of the cement paste with increasing ammonia content in fly ash.

The results presented in Section 3.1.2 showed that denitrified fly ash retarded the setting time of the fly ash cement paste. The effect of increasing ammonium content on the setting time of the fly ash cement paste is shown in Figure 9. It was observed that when the additional ammonia content was less than 1000 mg/kg, there was no obvious trend that governed the initial setting time of the samples as the ammonia content increased. When more than 1000 mg/kg of ammonia was added, the initial setting time of the fly ash cement paste was increased as the amount of additional ammonia increased. In addition, when the additional ammonia content was greater than 400 mg/kg, the final setting time of the fly ash cement paste was increasingly prolonged with the additional ammonia content of the fly ash. Overall, increasing the ammonia content of the denitrified fly ash would prolong the cement paste’s initial and final setting times. This was related to the consumption of OH^−^ by NH_4_^+^ and H^+^ from NH_4_HSO_4_, which decreased the alkalinity of the paste.

#### 3.2.3. Compressive Strength

**Figure 10:**
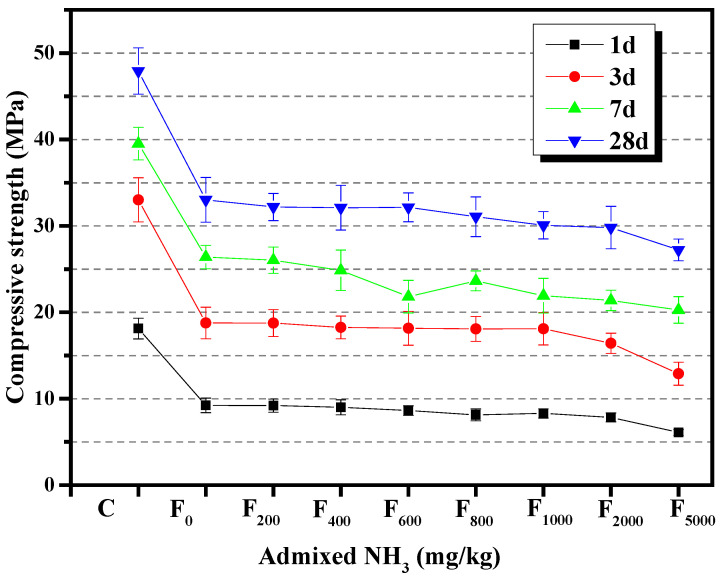
The effect of increasing ammonia content in fly ash on the compressive strength of the resultant cement paste.

The results presented in Section 3.1.3 showed that NH_4_HSO_4_ reduced the compressive strength of the fly ash cement paste. The effect of increasing ammonia content in denitrified fly ash on the compressive strength of the fly ash cement paste is shown in Figure 10. It could be seen that the compressive strength of the fly ash cement paste after 1, 3, 7, and 28 days exhibited a downward trend as the ammonia content of the denitrified fly ash increased. When the additional ammonia content of the fly ash was more than 1000 mg/kg, the compressive strength decreased significantly. When the additional ammonia content was 5000 mg/kg, the compressive strength of fly ash cement paste after three days was 28.75% lower than the same value recorded when only 1000 mg/kg of additional ammonia was added. This suggests that the compressive strength of the fly ash cement paste decreased as the ammonia content of the denitrified fly ash increased.

#### 3.2.4. Drying Shrinkage

**Figure 11:**
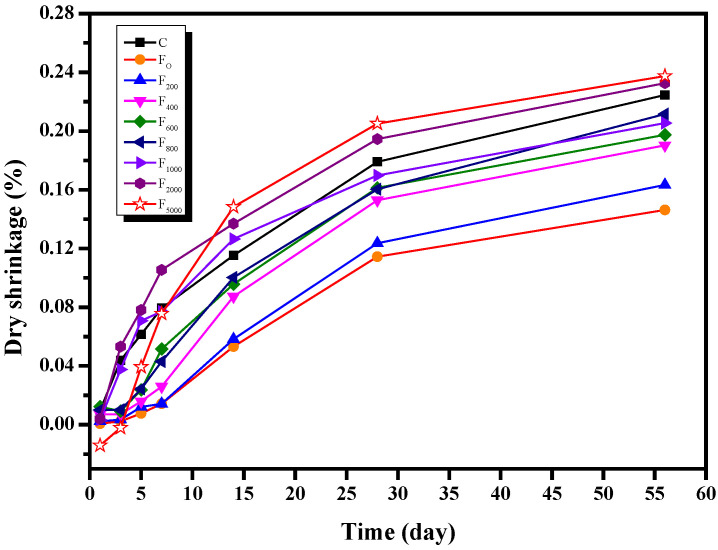
The effect of increasing fly ash ammonia content on the drying shrinkage of cement paste.

According to Section 3.1.4, NH_4_HSO_4_ increases the drying shrinkage of the fly ash cement paste. The drying shrinkage of the fly ash cement paste with low ammonia content is unknown. The effect of increasing ammonia content on the drying shrinkage of the fly ash cement paste is shown in Figure 11. Compared to pure cement paste, the fly ash with no additional ammonia content can reduce the drying shrinkage experienced by the fly ash cement paste in the early stages. As the ammonia content of denitrified fly ash increased, the drying shrinkage of the cement paste also increased. When the amount of ammonia added was less than or equal to 800 mg/kg, the fly ash still reduced the drying shrinkage of the cement paste compared to pure cement paste. However, when more than 800 mg/kg of ammonia was added, the dry shrinkage of the fly ash cement paste was increased compared to pure cement paste; above these values, the dry shrinkage of the fly ash cement paste increased as the ammonia content increased. In addition, after 56 days, the drying shrinkage of samples with an additional ammonia content of 5000 mg/kg was 62.4% greater compared to the fly ash cement paste without any additional ammonia and 5.8% greater compared to pure cement paste. These observations could be explained by the fact that during the reaction of NH_4_HSO_4_ with cement and water, the release of ammonia removed a portion of the available water while also increasing the opening porosity of the hardened fly ash cement paste; this increased the contact area between the specimen and the external environment, which accelerated the loss of water and increased drying shrinkage. Furthermore, the volume expansion experienced by the one-day-old fly ash cement paste with an additional ammonia content of 5000 mg/kg could be explained by the high ammonia content, which accelerated the early release of ammonia and resulted in volume expansion.

#### 3.2.5. Pore Structure

Previous studies have suggested that the pore structure of cement concrete can be improved and that the volume of large pores in the paste decreases when fly ash is added into cement concrete as the mineral admixture; this is beneficial to the late strength of the fly ash cement paste [20,24]. The results described in Section 3.1.4 and Section 3.2.4 indicate that the NH_4_HSO_4_ in denitrified fly ash led to the release of ammonia, increased the drying shrinkage of the fly ash cement paste, and led to volume expansion, which may affect the internal structure of the cement after hardening, as well as affect the pore structure of the fly ash cement paste. Thus, mercury injection was used to study the effect of increasing ammonia content in denitrified fly ash on the pore structure of the resultant cement paste.

**Figure 12:**
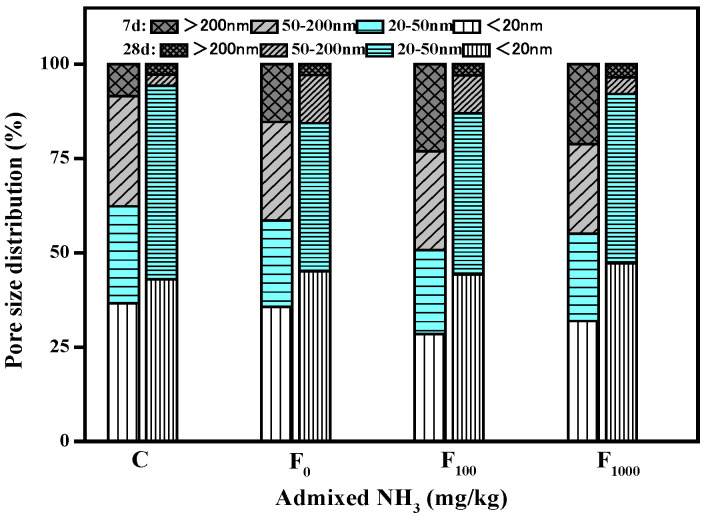
The effect of increasing ammonia content in fly ash on the pore size distribution of the cement paste.

The changes in the porosity and pore size distribution of the fly ash cement paste with increasing ammonia content is shown in Figure 12. As the ammonia content increased, the number of large pores in the cement paste increased while the number of small pores decreased. This was especially significant in the number of pores with sizes greater than 200 nm; the number of pores larger than 200 nm after 7 and 28 days in samples with 1000 mg/kg of additional ammonia content increased by 5.9% and 0.6%, respectively, compared to samples without any additional ammonia. Considering that NH_4_HSO_4_ in denitrified fly ash led to the release of ammonia gas, as the ammonia content increased, the porosity and the number of large pores in the fly ash cement paste also increased, which adversely affected the strength and volume shrinkage of the cement paste. The results of the pore structure experiments were consistent with the previous results obtained regarding the compressive strength and drying shrinkage of the cement pastes.

## 4. Conclusions

(1) Compared with cement slurry without NH_4_HSO_4_, when the NH_4_HSO_4_ content was 2%, the fluidity of the paste decreased by 17.0%, the initial setting time lengthened by 12.8%, and the compressive strength of 1 d decreased by 22.8%. When the NaHSO_4_ content was 2%, the fluidity of the paste decreased by 2.7% and initial setting time lengthened by 8.5%. The 1 d’s compressive strength increased by 1.3%, and it was found that the NH^4+^ in the by-product of denitrified fly ash was the main reason that affected the performance of the fly ash cement paste, while NH^4+^ obviously reduced the fluidity of the fly ash cement paste and retarded the setting time of the fly ash cement paste.
(2) Through hydration heat and TG analysis, NH^4+^ will reduce the peak and total heat release of the fly ash cement paste during hydration and affect the calcium hydroxide hydrate content.
(3) When the content of NH_4_HSO_4_ was 1000 mg/kg, the number of large pores in the net paste of the fly ash cement increased by 5.9% and 0.6% at 7d and 28d, respectively, compared with that of the net paste of the fly ash cement with the content of NH_4_HSO_4_ being 0 mg/kg. Therefore, NH^4+^ will increase the porosity of the cement stone and the number of macropores in the fly ash cement paste, thereby reducing the strength of the fly ash cement paste and increasing the drying shrinkage of the fly ash cement paste. In practical engineering, ammonia content in denitrified fly ash should be strictly controlled.

## Figures and Tables

**Table 1:** Chemical composition of fly ash and cement (%).

	SiO_2_	Al_2_O_3_	Fe_2_O_3_	CaO	SO_3_	TiO_2_	K_2_O	Na_2_O	MgO	LOI	Other
Fly ash	49.16	27.84	7.26	4.97	2.57	1.72	1.66	1.33	-	4.32	3.47
Cement	23.22	5.16	2.53	61.32	2.95	-	-	-	2.19	2.92	2.63

**Table 2:** Compound ratios used in the preparation of fly ash containing different sulfates.

	Fly Ash (g)	NH_4_HSO_4_ (g)	NaHSO_4_ (g)
F-0	300	0.00	0.00
F-NH_4_HSO_4_	300	6.00	0.00
F-NaHSO_4_	300	0.00	6.26

Note: NH_4_HSO_4_ was mixed with 2% fly ash mass. Based on the relative molecular mass of the NH_4_HSO_4_ mixture, the same number of moles of NaHSO_4_ was used to prepare the other mixtures.

**Table 3:** Compound ratios used to prepare samples with different NH_4_HSO_4_ concentrations.

	Cement (g)	Fly Ash (g)	Water (g)	Additional NH_4_HSO_4_ (mg/kg)	Total Ammonia (mg/kg)
C	500	0	200	0	0
F_0_	350	150	200	0	105
F_200_	350	150	200	200	305
F_400_	350	150	200	400	505
F_600_	350	150	200	600	705
F_800_	350	150	200	800	905
F_1000_	350	150	200	1000	1105
F_2000_	350	150	200	2000	2105
F_5000_	350	150	200	5000	5105

Note: The additional NH_4_HSO_4_ is calculated based on fly ash.

## Data Availability

Not applicable.

## References

[1] Zhang C.Y., Wang S.X., Xing J., Zhao Y., Hao J.M. (2008). Current status and future projections of NOx emissions from energy related industries in China. Huanjing Kexue Xuebao/Acta Sci. Circumstantiae.

[2] Li X., Zhu P., Wang F., Wang L., Tsang S.C. (2008). Simultaneous Catalytic Reductions of NO and SO_2_ by H_2_ over Nickel-Tungsten Catalysts. J. Phys. Chem. C.

[3] Wu B., Wang S., Fang Z. (2006). Flue Gas Denttrification Technologies and Analysis of Their Chemical Reactions. Therm. Power Gener..

[4] Larrimore L., Monroe L., Dodgen D. (1999). Characterization of Ammonia Effects on Ash and Evaluation of Removal Methods.

[5] Brendel G.F., Bonetti J.E., Rathbone R.F., Frey R.N. (2000). Investigation of Ammonia Adsorption on Fly Ash Due to Installation of Selective Catalytic Reduction Systems.

[6] Shou L., Hayes J., Cheng W., Wu C.Y., Townsend T., Vinson T., Schert J. (2014). Characterization of ammonia gas release from concrete added with ammoniated fly ash. Air Qual. Atmos. Health.

[7] Schert J., Townsend T., Wu C.Y. (2012). Identification of Potential Concerns Associated with FDOT Use of Ammoniated Fly Ash.

[8] Duan F., Chyang C.S., Zhang L.H., Chi Y.T. (2015). Effect of the molecular structure of nitrogen compounds on the pollutant formation in a bubbling fluidized-bed combustor. Energy.

[9] Chyliński F., Goljan A., Michalik A. (2021). Fly ash with ammonia: Properties and emission of ammonia from cement composites. Materials.

[10] Wu D. (2009). Analysis of Concrete Abnormal Phenomena Caused by Off-Grade Fly Ash. Coal Ash.

[11] He Y. (2018). Effects of Ammonia Release and Retention of Ammoniated Fly Ash on the Performance of Cement Concrete. Master’s Thesis.

[12] Tan X., Yang L., Rui O., Lin M., Kang M. (2016). Research on Application of Denitrated Fly Ash in Cement and Concrete. Coal Ash.

[13] Liu Y., Kong X., Wu K., Zhang S., Ji G., Liu C. (2022). Effect of the Ammonia Present in Fly Ash on the Properties of Fly Ash and Mortar. Water Power.

[14] Zheng X., Kong X., Liu C., Ji G., Zhu Z., Kang X., Wang H., Pei X. (2020). Effect of Ammonia Content in Denitrification Fly Ash on the Properties of Fly Ash and Mortar. Cement.

[15] Liu Y., Wang K., Guo H., Wu H., Wang H. (2020). Experimental Study on the Effect of Ammonia in Fly Ash on the Filling Paste Performance. Coal Eng..

[16] Qin H., Liu Z., Jia X., Wang Z. (2020). Effect and Mechanism of Denitrification Fly Ash on Performance of Cement Concrete. Fly Ash Compr. Util..

[17] Zhang Y. (2016). Study on the Effect of Selective Catalytic Reduction Denitration (SCR) on the Properties of Fly Ash. Master’s Thesis.

[18] Wang L. (2017). Study on the Mechanism and Control Technology of Denitrification and Desulphurization Fly Ash on Cement Performance. Master’s Thesis.

[19] Qin L., Gao X., Li Q. (2019). Influences of coal fly ash containing ammonium salts on properties of cement paste. J. Environ. Manag..

[20] Qian J.S. (2002). Characteristics of Fly Ash and Fly Ash Concrete.

[21] Wang Z., Wang Z., Sun H., Zhang Y., Wen C., Zhang Y., Tang Z. (2016). Effect of Denitration on Properties of Fly Ash as Mineral Admixtures. Bull. Chin. Ceram. Soc..

[22] Sun H., Qian J., Yang S., Xiong Q., Chen X. (2017). Effect of Alkali Sulfates in Clinker on Early Setting Behavior of Portland Cement. J. Chin. Ceram. Soc..

[23] Nguyen T.H.Y., Tran V.M., Pansuk W., Cao N.T. (2021). Electrochemical chloride extraction on reinforced concrete contaminated external chloride: Efficiencies of intermittent applications and impacts on hydration products. Cem. Concr. Compos..

[24] Qian J., Wu C., Wang Z. (2001). Mineral composition of fly ash (I). Fly Ash Compr. Util..

